# Case Report: A giant gallstone causing cholecystoduodenal fistula with Bouveret syndrome and acute pancreatitis

**DOI:** 10.3389/fmed.2026.1813702

**Published:** 2026-04-30

**Authors:** Xiong Fan, Qianxiong Huang

**Affiliations:** Department of Gastroenterology, Yueyang Central Hospital, Yueyang, China

**Keywords:** acute pancreatitis, Bouveret syndrome, duodenal obstruction, endoscopic lithotripsy, giant gallstone

## Abstract

**Introduction and importance:**

Bouveret syndrome is a rare form of gallstone ileus characterized by gastric outlet obstruction secondary to impaction of a gallstone that has migrated into the gastrointestinal tract through a cholecystoduodenal fistula. It predominantly occurs in elderly patients with multiple comorbidities. Cases involving giant gallstones complicated by acute pancreatitis are exceedingly uncommon and pose significant therapeutic challenges.

**Case presentation:**

A 72-year-old woman was admitted with a 6-day history of persistent right upper quadrant abdominal pain and distension, accompanied by nausea, vomiting, and constipation. Her medical history was notable for diabetes mellitus, hypertension, prior cerebral infarction, and chronic renal insufficiency.

**Clinical findings and investigations:**

Physical examination revealed marked tenderness in the right upper quadrant with a positive Murphy’s sign. Laboratory evaluation demonstrated leukocytosis and markedly elevated serum amylase levels, consistent with acute pancreatitis. Abdominal computed tomography (CT) showed cholelithiasis, cholecystitis, pneumobilia, and findings suggestive of a cholecystoduodenal fistula. Endoscopy revealed a giant gallstone measuring approximately 3.5 × 6.0 cm impacted in the descending portion of the duodenum, resulting in complete obstruction.

**Intervention and outcomes:**

Under general anesthesia, the stone was successfully fragmented and removed in a single endoscopy session using electrohydraulic lithotripsy combined with a retrieval basket and snare under C-arm fluoroscopic guidance. The procedure was completed under a single session of general anesthesia, restoring duodenal patency. Postoperatively, the patient received broad-spectrum intravenous antibiotics and supportive therapy, with significant symptom relief. She recovered uneventfully and was discharged on postoperative day 6, with no recurrence observed during three-month follow-up.

**Relevance and impact:**

This case highlights that, in elderly high-risk patients, endoscopic lithotripsy and stone extraction may serve as a safe, effective, and minimally invasive first-line treatment option for Bouveret syndrome caused by giant gallstones, particularly when complicated by acute pancreatitis, offering valuable clinical insight.

## Introduction

Bouveret syndrome is defined as gastric outlet obstruction caused by a gallstone that has migrated into the duodenum through a biliary-enteric fistula. It accounts for approximately 1%–3% of all cases of gallstone ileus (1, 2), and represents a rare cause of gastric outlet obstruction. Other rare causes of gastric outlet obstruction include gastroduodenal intussusception, which has been reported as an insidious complication following certain surgical procedures (3). The condition typically affects elderly individuals with multiple comorbidities (4). Endoscopy serves as both a valuable diagnostic and therapeutic modality for stone identification and removal (5). Cases involving giant gallstones complicated by acute pancreatitis are extremely rare and sparsely reported in the literature (6). Systemic inflammatory responses and pancreatic inflammation may further increase the technical difficulty of duodenal intervention, thereby making management more challenging. This case report is presented in accordance with the SCARE criteria (7).

## Case presentation

A 72-year-old woman presented with a 6-day history of persistent right upper quadrant abdominal pain and distension radiating to the shoulder and back, accompanied by nausea, vomiting, and constipation. On admission, her blood pressure was 160/95 mmHg. Physical examination revealed marked tenderness in the right upper quadrant, and Murphy’s sign was positive. Laboratory evaluation demonstrating leukocytosis with a white blood cell count of 13.33 × 10^9^/L and 84.70% neutrophils, hemoglobin of 109 g/L, and platelet count of 282 × 10^9^/L. C-reactive protein was elevated at 21.40 mg/L, indicating a systemic inflammatory response. Serum amylase was markedly elevated to 6624.1 U/L (reference range: 13.0–60.0 U/L), and urinary amylase was 13,905 U/L (reference range: 0–900 U/L), exceeding three times the upper limit of normal (ULN). These findings were consistent with acute pancreatitis. Serum lipase was also significantly elevated. Liver function tests including alanine aminotransferase (ALT), aspartate aminotransferase (AST), gamma-glutamyl transferase (GGT), and alkaline phosphatase (ALP) were all within normal limits, suggesting no significant biliary obstruction or cholestasis at presentation. Renal function showed elevated creatinine at 127.7 μmol/L, consistent with her known chronic renal insufficiency.

Non-contrast abdominal computed tomography (CT) revealed inflammatory changes of the gallbladder suggestive of cholecystitis, along with a suspected communication between the gallbladder and duodenum, highly indicative of a cholecystoduodenal fistula. No significant dilatation of the intrahepatic or extrahepatic biliary tree was observed. Multiple nodular hyperdense lesions were identified within the duodenal lumen, surrounded by inflammatory changes, consistent with ectopic gallstones. In addition, the pancreatic head appeared mildly enlarged, with peripancreatic and periduodenal fat stranding, consistent with imaging features of acute pancreatitis.

**FIGURE 1:**
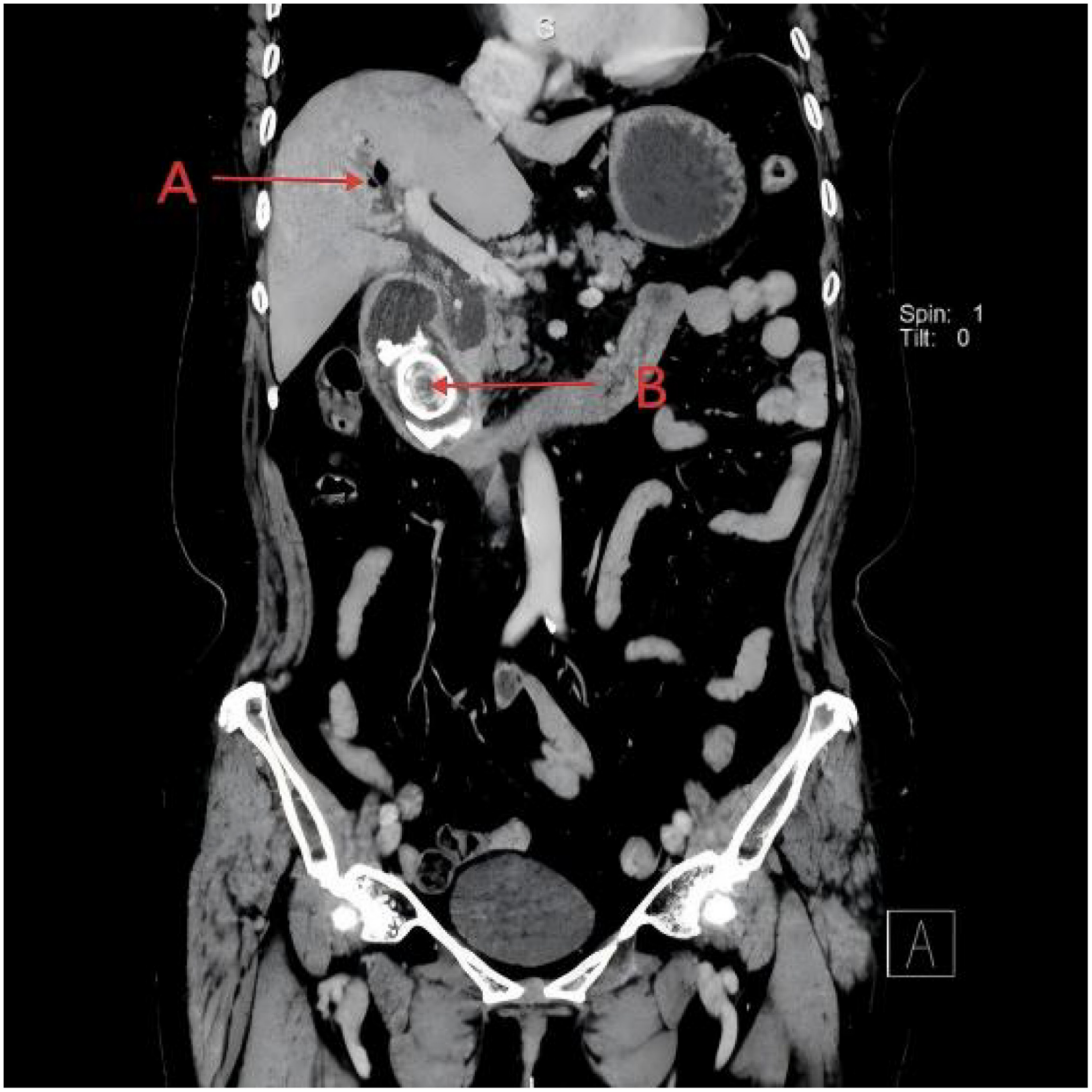
Coronal abdominal computed tomography (CT) showing a giant gallstone impacted in the descending duodenum, consistent with imaging features of Bouveret syndrome. Arrow A indicates pneumobilia. Arrow B indicates ectopic giant gallstone in the duodenum.

**FIGURE 2:**
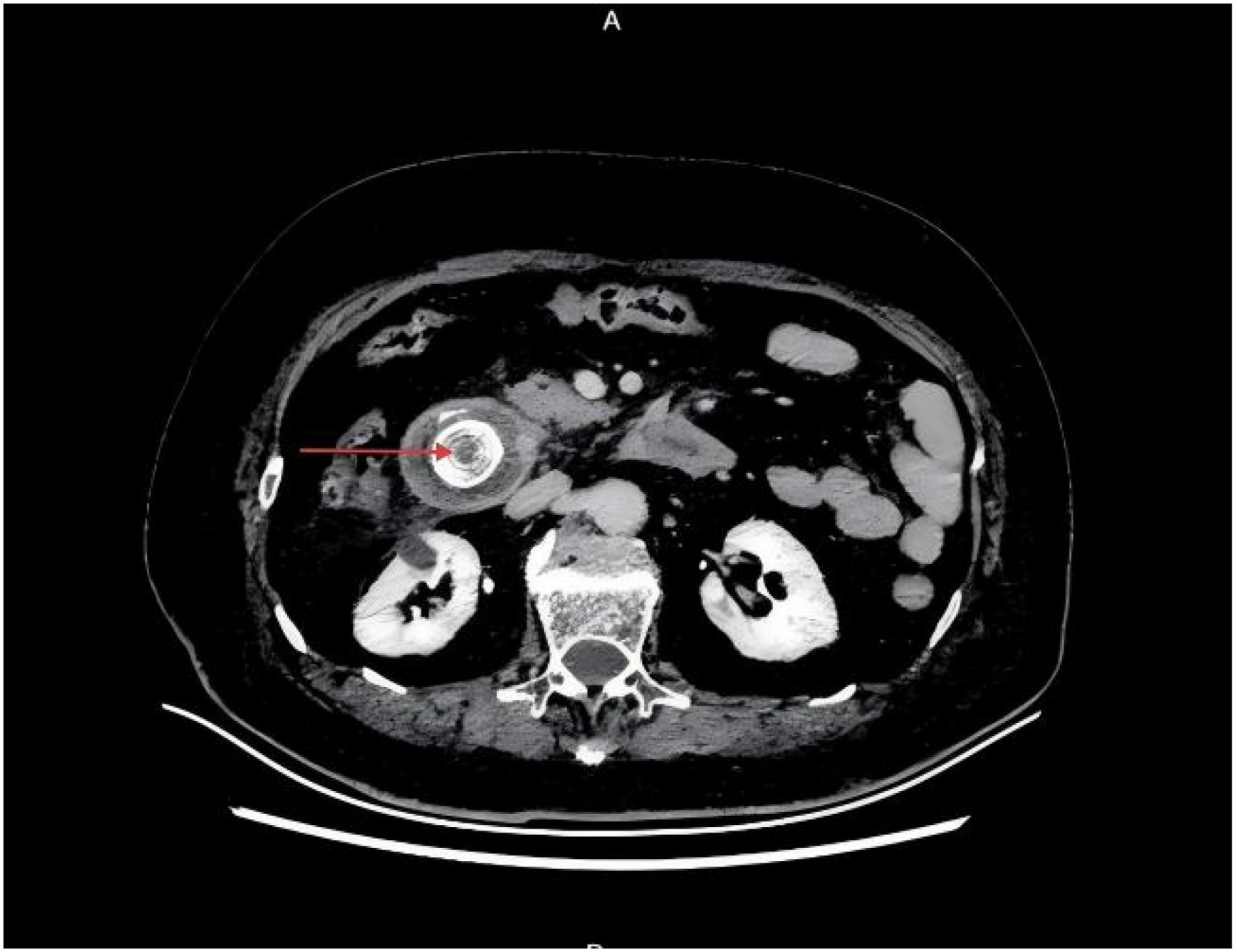
Axial abdominal computed tomography (CT) showing a ring-shaped hyperdense lesion within the duodenal lumen, indicating complete obstruction. Arrow indicates giant gallstone.

Pancreatitis severity was assessed using the BISAP scoring system, yielding a score of 3 (based on age > 60 years, elevated BUN > 25 mg/dL, and fulfillment of SIRS criteria), indicating a high risk for severe acute pancreatitis. Coronal reconstruction images demonstrated a giant gallstone impacted in the descending portion of the duodenum, consistent with the characteristic radiologic features of Bouveret syndrome (Figure 1, arrow indicating impacted stone). Axial CT images revealed a ring-shaped hyperdense lesion within the duodenal lumen, indicating complete obstruction caused by the impacted stone (Figure 2, arrow indicating obstructing stone).

The patient had multiple comorbidities, including diabetes mellitus, hypertension, previous cerebral infarction, chronic renal insufficiency, and prior bilateral rib fractures, placing her at high operative risk. Based on CT findings demonstrating no choledocholithiasis or biliary ductal dilatation, together with the temporal relationship between stone impaction and symptom onset, our diagnostic reasoning was that the acute pancreatitis was most likely secondary to mechanical compression of the ampulla of Vater by the impacted giant gallstone rather than passage of common bile duct stones. Given the high BISAP score and the likelihood of ongoing mechanical obstruction, urgent endoscopic intervention was prioritized to relieve both gastric outlet obstruction and ampullary compression, thereby preventing further clinical deterioration.

**FIGURE 3:**
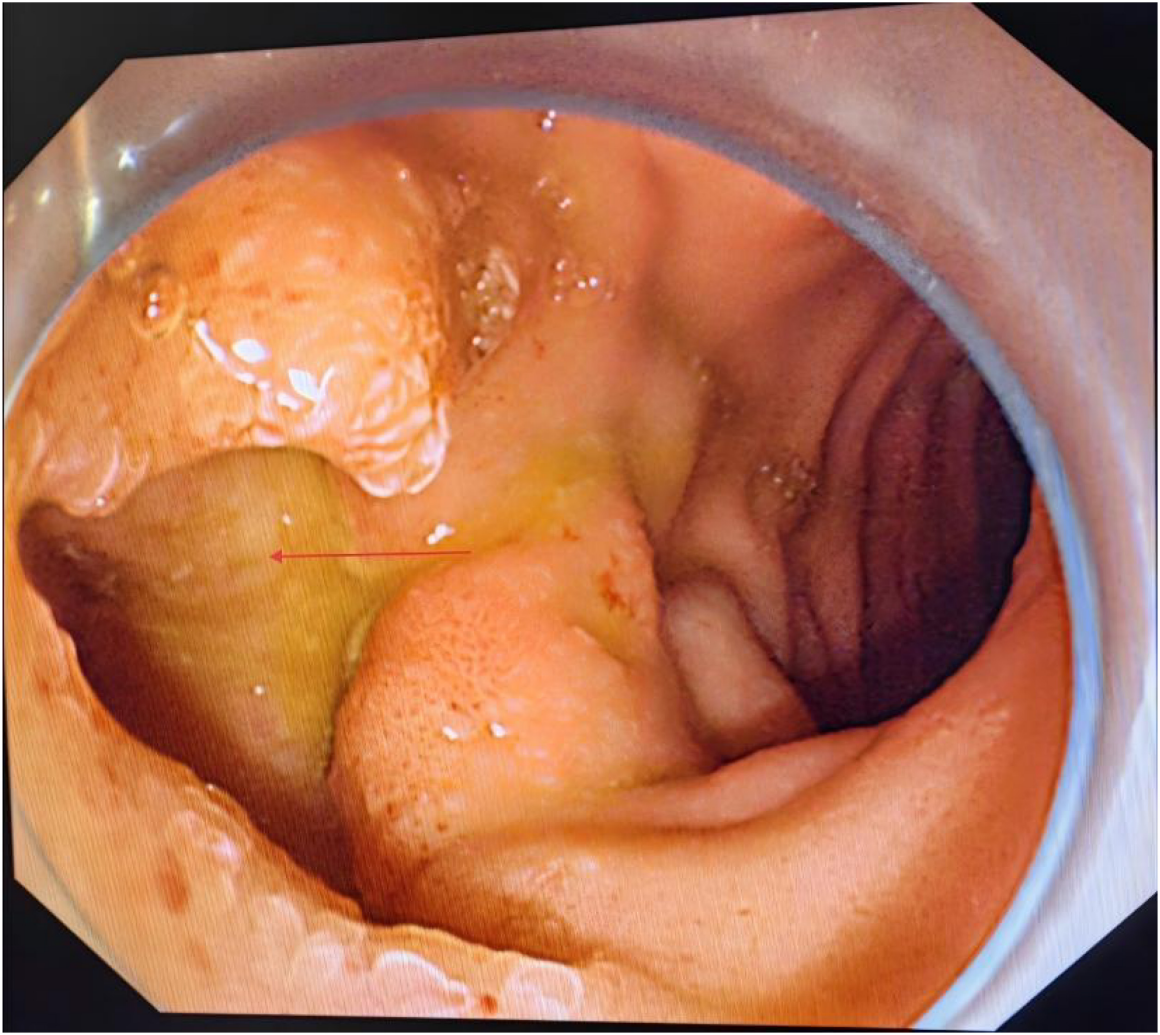
Endoscopic image demonstrating a cholecystoduodenal fistula, providing the anatomical pathway for gallstone migration into the duodenum. Arrow indicates fistulous opening between the biliary system and the duodenum.

**FIGURE 4:**
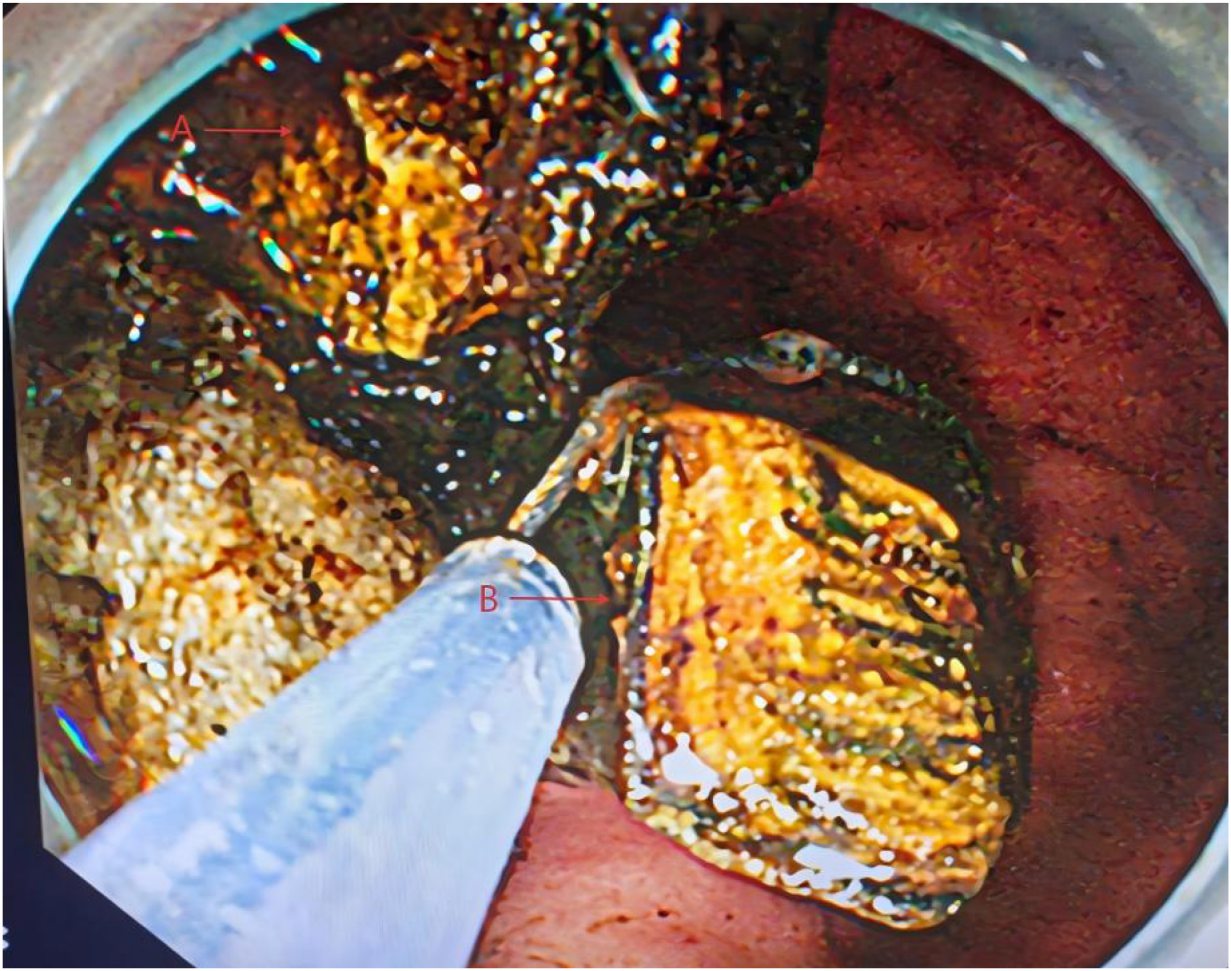
Endoscopic retrieval of fragmented gallstone pieces using a stone basket. Arrow A indicates fragmented gallstone. Arrow B indicates electrohydraulic lithotripsy probe.

Upper gastrointestinal endoscopy was performed under general anesthesia with endotracheal intubation. Endoscopy revealed a fistulous opening approximately 2.5 × 3.0 cm on the greater curvature side of the duodenal bulb, covered with whitish exudate, consistent with a cholecystoduodenal fistula (Figure 3, arrow indicating fistula opening). A giant stone was visualized in the descending duodenum, completely obstructing the lumen. Under guidewire assistance, a balloon catheter was advanced beyond the stone. C-arm fluoroscopy confirmed a filling defect measuring approximately 3.5 × 6.0 cm. Electrohydraulic lithotripsy was performed using a high-frequency pulse system with adjustable energy levels (0.1–0.4 J), pulse duration < 2 μs, and frequency > 30 Hz, enabling efficient stone fragmentation. The device provides a maximum output voltage of 4 kV and is designed for endoscopic use, ensuring precise and controlled lithotripsy in the duodenal lumen. The lithotripsy was applied using energy settings of 90–100 J per pulse, with approximately 150–200 pulses delivered over a total procedure time of 7 h to fragment the stone. The fragments were removed in stages using a retrieval basket and snare (Figure 4, arrows indicating stone fragments). Duodenal patency was successfully restored following the procedure, which was completed in one session under a single sequence of general anesthesia.

Electrohydraulic lithotripsy was performed using a high-frequency pulse system with adjustable energy levels (0.1–0.4 J), pulse duration < 2 μs, and frequency > 30 Hz, enabling efficient stone fragmentation. The device provides a maximum output voltage of 4 kV and is designed for endoscopic use, ensuring precise and controlled lithotripsy in the duodenal lumen. Under guidewire assistance, a balloon catheter was advanced beyond the stone. C-arm fluoroscopy confirmed a filling defect measuring approximately 3.5 × 6.0 cm. Electrohydraulic lithotripsy was then repeatedly applied to fragment the stone, and the fragments were removed in stages using a retrieval basket and snare (Figure 4). Duodenal patency was successfully restored following the procedure.

Postoperatively, the patient received broad-spectrum intravenous antibiotics, proton pump inhibitors, glycemic and blood pressure control, and supportive care. Her symptoms improved significantly. Repeat laboratory testing on postoperative day 4 demonstrated marked improvement in pancreatitis markers, with serum lipase decreasing to 263.8 U/L and urinary amylase to 1209.0 U/L, confirming resolution of acute pancreatitis following stone removal (Table 1). After resuming a light, low-fat diet without discomfort and restoration of normal bowel function, she was discharged on postoperative day 6 in stable condition. At 1-week follow-up, she reported no recurrence of symptoms, and she achieved full recovery during subsequent follow-up. Considering her advanced age, multiple comorbidities, and personal preference, elective cholecystectomy was not pursued.

**TABLE 1:** Laboratory findings before and 4 days after intervention.

Category	Parameter (unit)	Pre-treatment	Postoperative day 4	Reference range	Interpretation
Pancreatitis-related markers	Lipase (U/L)	6624.1	263.8	13.0–60.0	High
	Urinary amylase (U/L)	13905.0	1209.0	0–900.0	High
	C-reactive protein (mg/L)	21.4	12.6	0–6.0	High
Liver function markers	Total bilirubin (μmol/L)	10.1	–	≤23.0	Within normal limits
	Direct bilirubin (μmol/L)	4.1	–	1.7–6.8	Within normal limits
	Indirect bilirubin (μmol/L)	6.0	–	1.7–17.0	Within normal limits
	Alanine aminotransferase (U/L)	10.2	–	7.0–40.0	Within normal limits
	Aspartate aminotransferase (U/L)	20.4	–	13.0–35.0	Within normal limits
	Alkaline phosphatase (U/L)	119.3	–	40–130	Within normal limits
	Gamma-glutamyl transferase (U/L)	58.6	–	10–60	Within normal limits
	Total bile acids (μmol/L)	0.2	–	0–12.0	Within normal limits
	Total protein (g/L)	66.7	–	65.0–85.0	Within normal limits
	Albumin (g/L)	30.1	–	40.0–55.0	Low
	Globulin (g/L)	36.6	–	20.0–40.0	Within normal limits
	Albumin/Globulin ratio	0.8	–	1.2–2.4	Low
Renal function markers	Urea (mmol/L)	9.7	–	3.1–8.8	High
	Creatinine (μmol/L)	94.3	–	41.0–81.0	High
	Uric acid (μmol/L)	592.0	–	155.0–357.0	High
Electrolytes	Potassium (mmol/L)	2.86	–	3.5–5.3	Low
	Sodium (mmol/L)	140.6	–	137.0–147.0	Within normal limits
	Chloride (mmol/L)	98.5	–	99.0–110.0	Low
	Calcium (mmol/L)	2.17	–	2.11–2.52	Within normal limits
Lipid profile	Triglycerides (mmol/L)	1.04	–	0.56–1.70	Within normal limits
	Total cholesterol (mmol/L)	3.14	–	3.11–5.18	Within normal limits
	HDL-C (mmol/L)	0.61	–	1.04–1.55	Low
	LDL-C (mmol/L)	2.08	–	–	Within normal limits
	Non-HDL-C (mmol/L)	2.53	–	–	Within normal limits
Hematology	WBC (×10^9^/L)	13.33	10.58	3.5–9.5	High
	Neutrophils (%)	84.7	74.2	40–75	High → normal
	Lymphocytes (%)	8.9	13.4	20–50	Low
	Monocytes (%)	5.6	9.0	3–10	Within normal limits
	Neutrophils (×10^9^/L)	11.31	7.85	1.8–6.3	High
	Lymphocytes (×10^9^/L)	1.18	1.42	1.1–3.2	Within normal limits
	Monocytes (×10^9^/L)	0.74	0.95	0.1–0.6	High
	Platelets (×10^9^/L)	336	222	125–350	Within normal limits
	RBC (×10^12^/L)	4.33	3.49	3.8–5.1	Low
	Hemoglobin (g/L)	124	100	115–150	Low

## Discussion

Bouveret syndrome is a rare form of gallstone-related intestinal obstruction resulting from impaction of a gallstone in the pylorus or proximal duodenum following migration through a cholecystoduodenal fistula. It represents approximately 1%–3% of gallstone ileus cases and only 0.3%–0.5% of overall gallstone-related complications (8). Because its clinical presentation is often nonspecific, including nausea, vomiting, and upper abdominal pain, early diagnosis relies heavily on imaging findings. The classic radiologic features include pneumobilia, an ectopic gallstone within the gastrointestinal lumen, and evidence of a biliary-enteric fistula.

In the present case, the stone measured approximately 3.5 × 6.0 cm, exceeding the size of most previously reported stones and meeting the criteria for a giant gallstone (9, 10). Most reported cases of Bouveret syndrome involve stones measuring 2.0–3.0 cm in diameter, and stones exceeding 5.0 cm in any dimension remain exceptionally rare. The present case is distinctive in that it involved a 6.0 cm stone complicated by acute pancreatitis, a combination seldom documented in the literature. Furthermore, successful endoscopic management in a single session under one-time anesthesia distinguishes this case from many previously reported instances requiring multiple interventions or conversion to surgery. Impaction of such a large stone in the descending portion of the duodenum led to complete gastric outlet obstruction. Notably, the condition was further complicated by acute pancreatitis, as evidenced by markedly elevated serum amylase levels and peripancreatic inflammatory changes on CT imaging. The absence of biliary ductal dilatation, choledocholithiasis, and cholestatic liver enzyme elevation, combined with the marked improvement in amylase levels following stone removal, strongly suggests that the pancreatitis was caused by direct mechanical compression of the ampulla of Vater by the impacted giant stone rather than by passage of biliary stones. This pathophysiologic mechanism has been rarely described in the literature and represents a unique feature of this case. The coexistence of duodenal obstruction and acute pancreatitis likely reflects local inflammatory extension and mechanical compression near the ampullary region, which may exacerbate pancreatic ductal obstruction. This dual pathology substantially increased the complexity of clinical management.

Patients with Bouveret syndrome are typically elderly and medically fragile, making surgical intervention associated with considerable morbidity and mortality (11). Traditional surgical strategies include enterolithotomy with or without cholecystectomy and fistula repair; however, these procedures carry significant operative risk in high-risk populations. In recent years, advances in therapeutic endoscopy have expanded minimally invasive options, including mechanical lithotripsy, electrohydraulic lithotripsy, and laser lithotripsy. Nevertheless, the success rate of endoscopic management decreases significantly with increasing stone size, and giant stones often require multiple treatment sessions (12). In the present case, electrohydraulic lithotripsy combined with basket and snare extraction under fluoroscopic guidance successfully relieved the obstruction without procedure-related complications. This approach avoided emergency surgery and its associated risks, highlighting the feasibility and minimally invasive advantages of endoscopic lithotripsy in carefully selected high-risk patients. However, long-term surveillance remains important, particularly in patients who do not undergo definitive fistula repair.

One important consideration in the present case is the relatively long endoscopic procedure time of approximately 7 h under general anesthesia, which is notably longer than the 1–2 h typically reported in prior endoscopic cases involving smaller gallstones (5, 13). Compared with endoscopic treatment, open or laparoscopic surgery for Bouveret syndrome is generally completed within a shorter operative time, although duration varies significantly with stone size, impaction, and additional procedures, and can extend substantially in complex cases (e.g., up to 460 min with dense adhesions) (14). Nevertheless, procedure time alone should not dictate management (15). In this elderly patient with multiple comorbidities, the patient and family preferred endoscopic therapy to avoid surgical trauma; despite the prolonged duration, complete stone removal was achieved in one session, with rapid recovery and discharge on postoperative day 6 (16). As summarized in Table 2, endoscopic approaches may offer net benefits in selected high-risk cases (17).

**TABLE 2:** Comparison of endoscopic and surgical approaches for Bouveret syndrome.

Approach	Advantages	Disadvantages
Endoscopic treatment	Minimally invasive; no external incision; suitable for high-risk or frail patients; potentially shorter overall recovery if successful	Procedure time may be prolonged (especially with giant or impacted stones); technically demanding; risk of incomplete clearance or need for further intervention
Surgical approach (open or laparoscopic)	Generally shorter operative time; high success rate for stone removal; allows direct visualization and management of associated findings	More invasive; greater surgical trauma; higher perioperative risks in elderly patients with comorbidities; longer recovery period

Despite successful stone extraction, the cholecystoduodenal fistula remained patent postoperatively. The long-term risks associated with a persistent fistula include recurrent cholangitis, gallstone ileus from residual gallbladder stones, and chronic biliary reflux into the gastrointestinal tract. However, given the patient’s advanced age, multiple comorbidities including chronic renal insufficiency and prior cerebral infarction, significant frailty, and personal preference to avoid further surgery, elective cholecystectomy with fistula repair was not pursued. Close clinical follow-up was arranged to monitor for potential complications. At 3-month follow-up, the patient remained asymptomatic with no evidence of recurrent biliary symptoms or complications, supporting a conservative approach in this high-risk individual. Long-term surveillance remains important, particularly in patients who do not undergo definitive fistula repair.

Overall, this case emphasizes that endoscopic lithotripsy may serve as an effective first-line strategy for giant gallstone-induced Bouveret syndrome, even when complicated by acute pancreatitis, provided that careful patient selection and multidisciplinary assessment are undertaken.

## Conclusion

Endoscopic management of duodenal obstruction caused by giant gallstones in high-risk elderly patients can achieve complete resolution of Bouveret syndrome and may be considered an effective first-line therapeutic alternative.

## Data Availability

The data analyzed in this study is subject to the following licenses/restrictions: the dataset is not publicly available due to patient privacy and institutional restrictions but is available from the corresponding author upon reasonable request. Requests to access these datasets should be directed to 80415179@qq.com.
